# Supplementary material for: The Pseudogymnoascus destructans Proteome Under Copper Stress Conditions
Source: J Fungi (Basel). 2026 Apr 27;12(5):318. doi: 10.3390/jof12050318 (PMC13208438; doi:10.3390/jof12050318)
Supplement: Supplementary file 1 [file jof-12-00318-s001.zip › jof-4228326-Supplemental File S1.pdf]

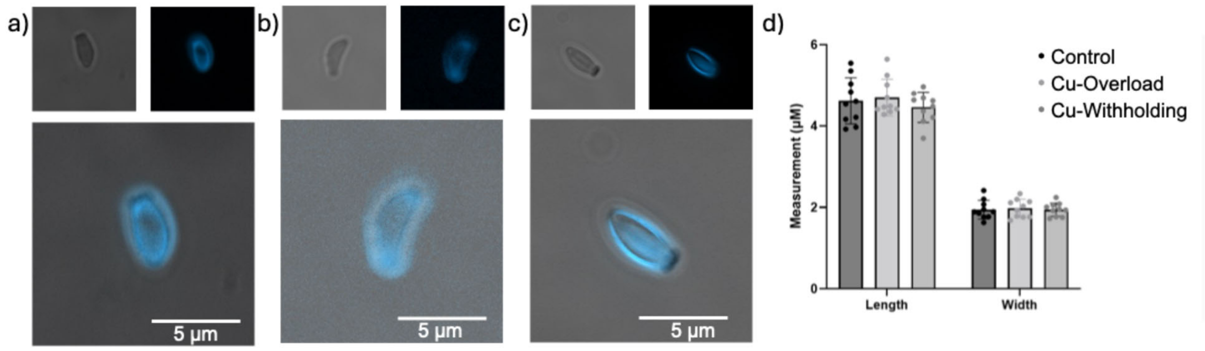

Supplemental Figure S1. Microscopy images of *P. destructans* spores isolated from Control, Cu-Overload, and Cu-Withholding growth conditions. A-C images of *P. destructans* Cells imaged under bright field (top left), Calcofluor-white (top right), and merged (bottom). Samples were cultured under (a) Control, (b) Cu-Overload, (c) Cu-Withholding growth conditions. (d). Graph displaying spore morphology parameters under different growth conditions, n = 10.
