# Supplementary material for: The Pseudogymnoascus destructans Proteome Under Copper Stress Conditions
Source: J Fungi (Basel). 2026 Apr 27;12(5):318. doi: 10.3390/jof12050318 (PMC13208438; doi:10.3390/jof12050318)
Supplement: Supplementary file 1 [file jof-12-00318-s001.zip › jof-4228326-Supplemental File S2.pdf]

Supplemental Table S1. Table of serum products used for the detection of *Pd* DAPs responding to Cu-withholding stress.

Serum Identifier	***Pd P*rotein Target**	**Peptides/Protein**	**Western Blot Dilution Ratio**
TSU8[1]	VC83_00191	KARQEARWLDCEMHRRYC; GKPGLRERVALHKDAKC	1:500
TSU9	VC83_04814	MSHSMGHGDHDASAARC; YDAALLKRRDELPHEELAC	1:1000
TSU12	VC83_01836	Recombinant full-length protein, VC83_01836, Plasmid pAS33	1:1000
TSU13/14	VC83_01837	Recombinant full-length protein, VC83_01837, Plasmid pAS32	1:1000
TSU15	VC83_01834	CNFLLPKDEVDGIEYS; CPIARSDSATREEEAK	1:1000
TSU17/18	VC83_01835	CGRSEKGQVEGGSNSY; CLVKRAASGKIITPED	1:1000

For peptide fragments, the reactive cystine used for KLH conjugation is underlined. [1] As previously reported by Anne, S.; Friudenberg, A.D.; Peterson, R.L. Characterization of a High-Affinity Copper Transporter *CTR1a* in the White-Nose Syndrome Causing Fungal Pathogen *Pseudogymnoascus destructans*. *J. Fungi* **2024**, *10*, 729.
